# Robust and Latch-Up-Immune LVTSCR Device with an Embedded PMOSFET for ESD Protection in a 28-nm CMOS Process

**DOI:** 10.1186/s11671-020-03437-3

**Published:** 2020-11-11

**Authors:** Ruibo Chen, Hongxia Liu, Wenqiang Song, Feibo Du, Hao Zhang, Jikai Zhang, Zhiwei Liu

**Affiliations:** 1 Key Laboratory for Wide-Band Gap Semiconductor Materials and Devices of Education, School of Microelectronics, Xidian University, Xi’an, 710071 China; 2 State Key Laboratory of Electronic Thin Films and Integrated Devices, University of Electronic Science and Technology of China, Chengdu, 610054 China

**Keywords:** ESD protection, Low-voltage-triggered SCR (LVTSCR), High holding voltage, Failure current, Turn-on resistance

## Abstract

Low-voltage-triggered silicon-controlled rectifier (LVTSCR) is expected to provide an electrostatic discharge (ESD) protection for a low-voltage integrated circuit. However, it is normally vulnerable to the latch-up effect due to its extremely low holding voltage. In this paper, a novel LVTSCR embedded with an extra p-type MOSFET called EP-LVTSCR has been proposed and verified in a 28-nm CMOS technology. The proposed device possesses a lower trigger voltage of ~ 6.2 V and a significantly higher holding voltage of ~ 5.5 V with only 23% degradation of the failure current under the transmission line pulse test. It is also shown that the EP-LVTSCR operates with a lower turn-on resistance of ~ 1.8 Ω as well as a reliable leakage current of ~ 1.8 nA measured at 3.63 V, making it suitable for ESD protections in 2.5 V/3.3 V CMOS processes. Moreover, the triggering mechanism and conduction characteristics of the proposed device were explored and demonstrated with TCAD simulation.

## Background

With the continuous miniaturization of semiconductor devices’ feature size, the damage induced by electrostatic discharge (ESD) in the integrated circuits (IC) has become a more serious problem. Moreover, the fabrication cost of the ESD protection device has been sharply increased due to the advanced process technology [1]. Therefore, the designs of high area efficiency and robustness ESD protection devices are of great values.

A silicon-controlled rectifier (SCR) device was verified to sustain a high ESD current with a small device dimension owing to the strongly positive feedback effect in its parasitic bipolar junction transistors [2]. But the SCR typically has a high trigger voltage (*V*_t1_) which exceeds the gate oxide breakdown voltage of the input stage in nanoscale CMOS technology. To reduce the *V*_t1_ of SCR, the modified lateral SCR (MLSCR) was reported for input ESD protection by inserting heavily doped n^+^ or p^+^ regions across the boundary of n-well and p-well [3, 4]. But the trigger voltage of the MLSCR is still greater than the breakdown voltages of output transistors in the CMOS output buffer. Therefore, it cannot provide an efficient output ESD protection alone.

To efficiently protect the CMOS output buffer, a low-voltage-triggered SCR (LVTSCR) has been proposed by inserting a short-channel NMOS (PMOS) device into the traditional SCR to realize a much lower trigger voltage [5–7], which is equivalent to the snapback trigger voltage of the inserted NMOS(PMOS) device. However, like the traditional SCR and MLSCR devices, the LVTSCR also suffers the hazards of latch-up due to its extremely low holding voltage (V_h_) of about 2 V [8]. Such a latch-up effect will result in a malfunction during normal operation and an incessant high current to destroy the IC [9].

There are several possible methods to improve the *V*_h_ of the LVTSCR [10–15]. The most common solution is to expand the base region of the SCR’s parasitic bipolar junction transistors (BJT) for decreasing the injection efficiency of their emitter–base junctions [10]. Adopting this solution, not only will the layout area of device increase, but the turn-on resistance (*R*_on_) should, too, which will further lead to drastic degradation of its failure current (*I*_t2_). Then, an optimized method was proposed by inserting a floating-n-well region in LVTSCR with less sacrifice on *I*_t2_ [11], but it also operates with a large *R*_on_ leading an exorbitant conduction voltage which exceeds the gate oxide breakdown voltage against a small current, thus reducing its effective ESD protection current. The method by adding the extra N-LDD/P-HALO layers in LVTSCR can also elevate the *V*_h_ [12], while such devices are only compatible for the specific process and cannot be widely used in ordinary CMOS processes. Moreover, a gate-to-ground NMOS-triggered LVTSCR (GGSCR) was reported in [13], which increases the *V*_h_ by leading the drain of the embedded NMOS to the anode, but it might cause the embedded NMOS to be damaged before the SCR conduction in a low current. Recently, the compound LVTSCR structures performing low *V*_t1_ as well as high *V*_h_ were demonstrated in [14, 15]. These compound structures are designed with high complexity and area requirements, thus limiting their application in advanced CMOS technology considering the design costs. Therefore, a robust, area-efficient, and latch-up-immune ESD protection device is highly desirable in advanced process ESD protection.

In this paper, a novel LVTSCR structure with an embedded PMOS transistor (EP-LVTSCR) is proposed for 2.5 V/3.3 V supply voltage applications. The proposed device was fabricated in a 28-nm CMOS process, and its electrical characteristic is verified through the measurement of transmission line pulse (TLP). The physics mechanisms of the proposed device are explored by technology computer-aided design (TCAD) simulations. As a result, the proposed structure possesses a higher holding voltage, a lower trigger voltage with a lower *R*_on_, and just a slight decrease in *I*_t2_ without any extra process step.

## Methods

**Fig. 1:**
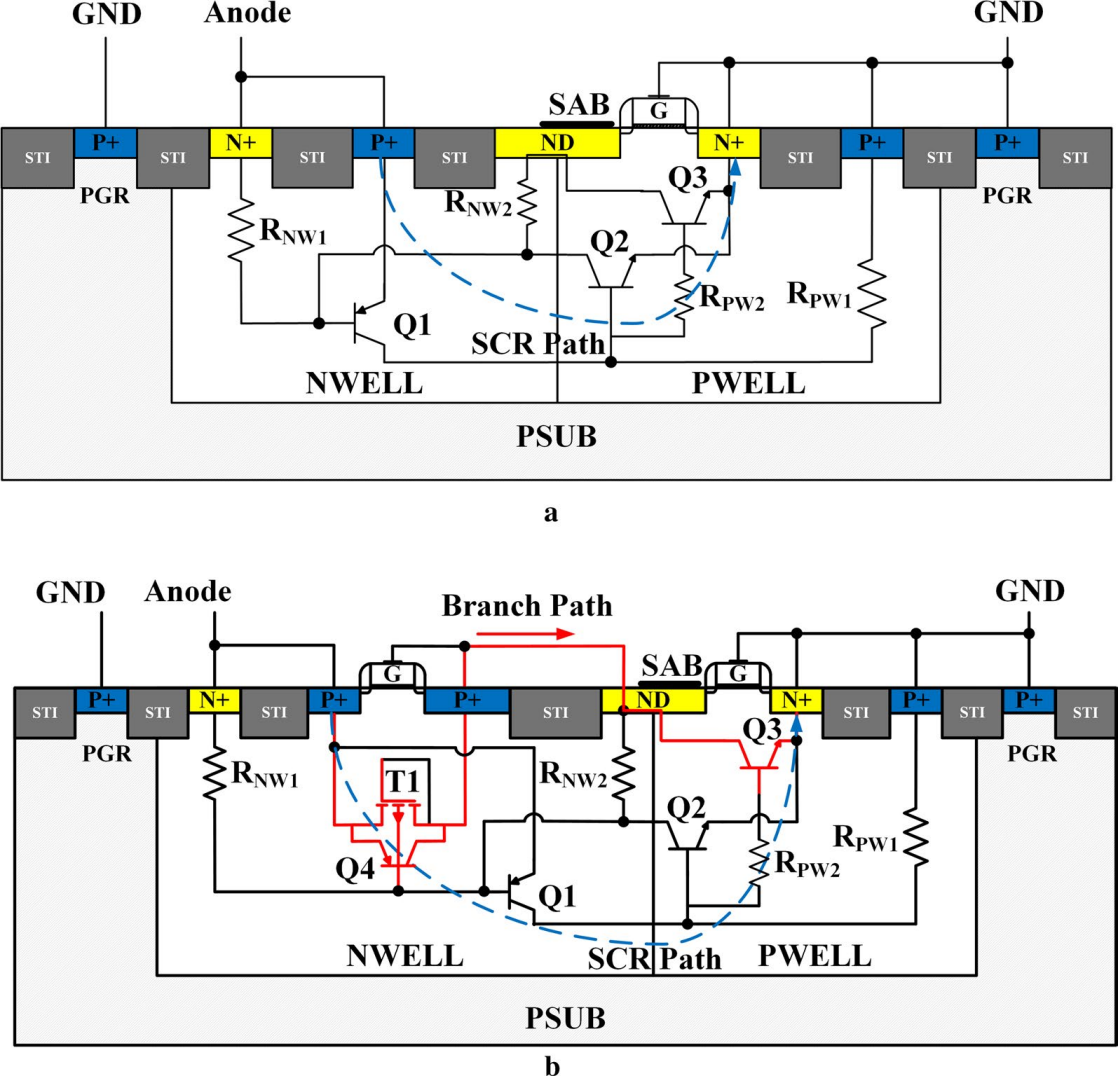
Cross-sectional view of **a** the conventional LVTSCR and **b** the proposed EP-LVTSCR

The conventional LVTSCR and the proposed EP-LVTSCR have been studied in this paper. The schematic cross-sectional views of the conventional LVTSCR and EP-LVTSCR are shown in Fig. 1a, b, respectively, whereas their equivalent circuits are depicted within the structures with the parasitic transistors and well resistors. In both devices, silicide block (SAB) layers are placed on the top of partial ND region which will induce ballast resistances by preventing silicide layers’ forming [16, 17]. In conventional LVTSCR, an NMOS transistor is inserted in PWELL with its drain (ND) setting across NWELL and PWELL, while its source and gate are connected to ground (GND) together, which can be seen in Fig. 1a. During ESD stress, the parasitic lateral p-n-p BJT (Q1) and the parasitic lateral n-p-n BJTs (Q2 and Q3) will be gradually turned on. In such a condition, the Q1 and Q2 transistors are coupled to constitute the SCR conduction path which will predominate the current discharge, where the SCR conduction path is indicated by the dashed line.

Compared to the conventional LVTSCR, EP-LVTSCR has an extra PMOS transistor (T1) embedded in the NWELL as its gate and drain are connected to ND with a metal, which is shown in Fig. 1b. When an ESD pulse is applied to the anode of the EP-LVTSCR, the reverse-biased N+/NWELL/N+/PWELL/P+ diode will conduct first if the zapping voltage is higher than the N+/PWELL breakdown voltage. Then, the holes/electrons generated by the avalanche multiplier effect will flow toward the cathode/anode, hence increasing the current density in NWELL/PWELL, and further elevate the drop potential across the NWELL/PWELL. Once the total voltage drop on *R*_NW1_ and *R*_NW2_, which is equivalent to the voltage drop between the source and gate (− *V*_gs_) of T1, overwhelms its threshold voltage (*V*_th_) of about 0.9 V, the T1 will be turned on. Subsequently, the parasitic lateral n–p–n transistors Q2 and Q3 will be triggered with reliance on the conduction of their emitter–base junctions. It is noticed that the conducting of T1 will lower the *R*_on_ across NWELL, and thus the Q2 and Q3 can be triggered in lower voltages. As the current continues to increase, the voltage drop on *R*_NW1_ rises to about 0.7 V and turns on the Q1 and the parasitic lateral p–n–p transistor of the PMOS (Q4). Finally, the SCR path turns on against the branch path conducting.

Several reports demonstrated that the holding voltage of SCR is mainly determined by the potential difference across the NWELL/PWELL depletion region *V*_dep_ [18–20], which is inversely proportional to the minority carriers (electrons/holes) injected into the depletion region. While the branch path of EP-LVTSCR can extract holes/electrons injected into the depletion region from the SCR path, thus elevating the *V*_h_ of EP-LVTSCR.

In order to further demonstrate the physics mechanism of EP-LVTSCR, TCAD simulation has been carried out, where the physics models such as mobility, recombination, thermodynamic, and effective intrinsic density were integrated and the mathematical methods like extrapolate, RelErrcontrol, and direct current computation were used. The ESD current modeling by 1.2 A pulses with 10 ns rise time was applied to the anodes of EP-LVTSCR and the conventional LVTSCR, respectively, where the substrate of the devices was regarded as the only heat sink and the ambient temperature was set as 300 K.

**Fig. 2:**
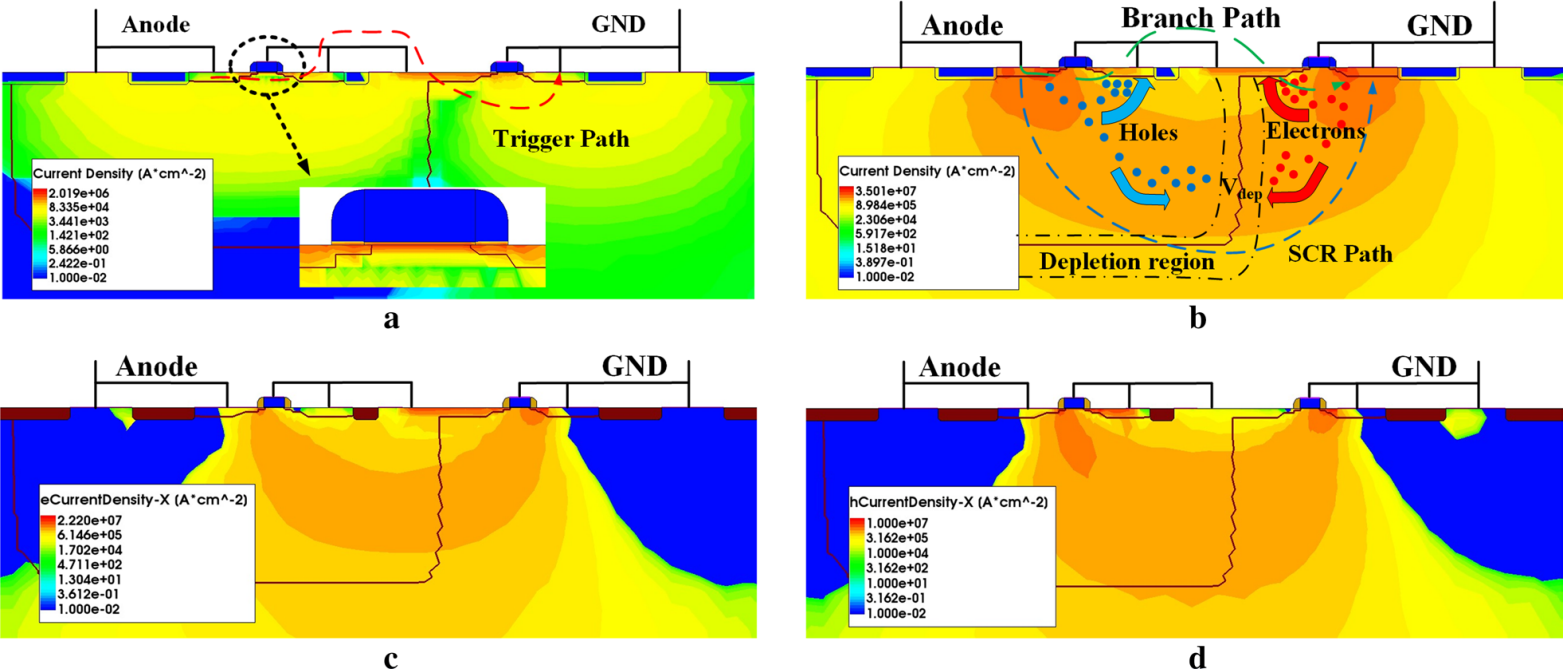
TCAD-simulated **a** total current density distribution at *t* = 500 ps, **b **total current density distribution at 5 ns, **c** horizontal electrons current density distribution at 5 ns and **d** horizontal holes current density distribution at 5 ns of the proposed EP-LVTSCR under a 1.2A-TLP stress

**Fig. 3:**
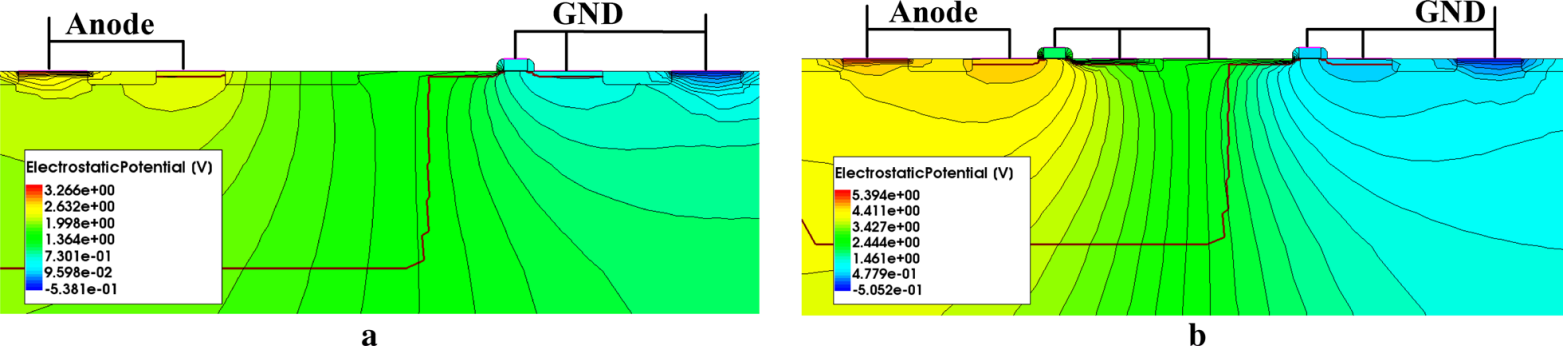
TCAD-simulated electrostatic potential distributions of **a** the proposed EP-LVTSCR and **b** the conventional LVTSCR at *t* = 5 ns under a 1.2A-TLP stress

The simulated results of the total current density distributions of EP-LVTSCR at 500 ps and 5 ns are shown in Fig. 2a, b, respectively. At 500 ps, the current density distribution concentrates on the T1 and the Q2 transistors, which indicates that the series T1/Q2 have turned on as a trigger path shown in Fig. 2a. When time rises to 5 ns, both the SCR path and the branch path have conducted as can be observed in Fig. 2b. At this time, part of the holes/electrons generated by P+/N+ are extracted from NWELL/PWELL to flow through the branch path, which are illustrated by the horizontal holes and electrons current density of the proposed structure shown in Fig. 2c, d. Further, the electrostatic potential distributions of the conventional LVTSCR and EP-LVTSCR at 5 ns are compared in Fig. 3a, b. Obviously, the potential peak inside the EP-LVTSCR is higher. These simulation results provide direct evidence for the above assumption of EP-LVTSCR’s higher holding voltage resulted from the branch path indicated in Fig. 1b.

## Results and discussion

**Fig. 4:**
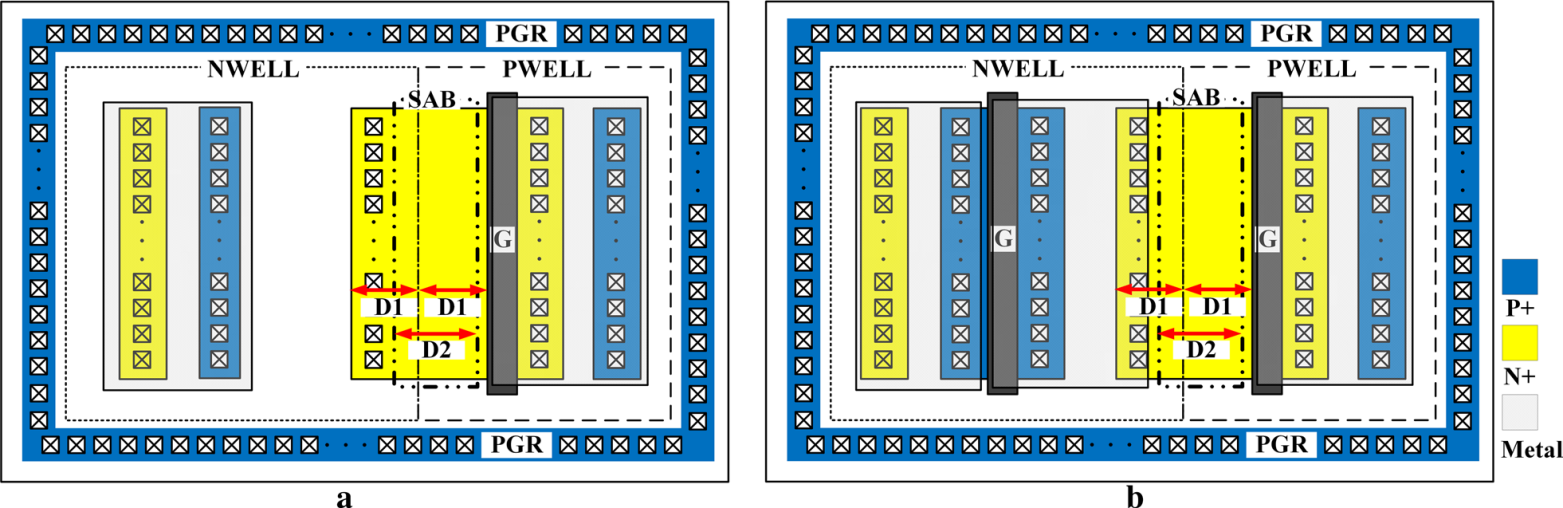
Layout topologies of **a** the conventional LVTSCR and **b** the proposed EP-LVTSCR

The conventional LVTSCR and EP-LVTSCR are implemented in a 28-nm 2.5 V/3.3 V logic CMOS process with the same width of 40 um, and their layout topologies are shown in Fig. 4a, b, respectively. To avoid the parasitic effects associated with the substrate, P-type guard rings (PGR) are employed in both structures, and each PGR is connected to GND [21]. The central axle of ND is aligned with the NWELL/PWELL junction, and the parameter D1 is used to describe half of the ND length, while D2 is the length of SAB region.

**Fig. 5:**
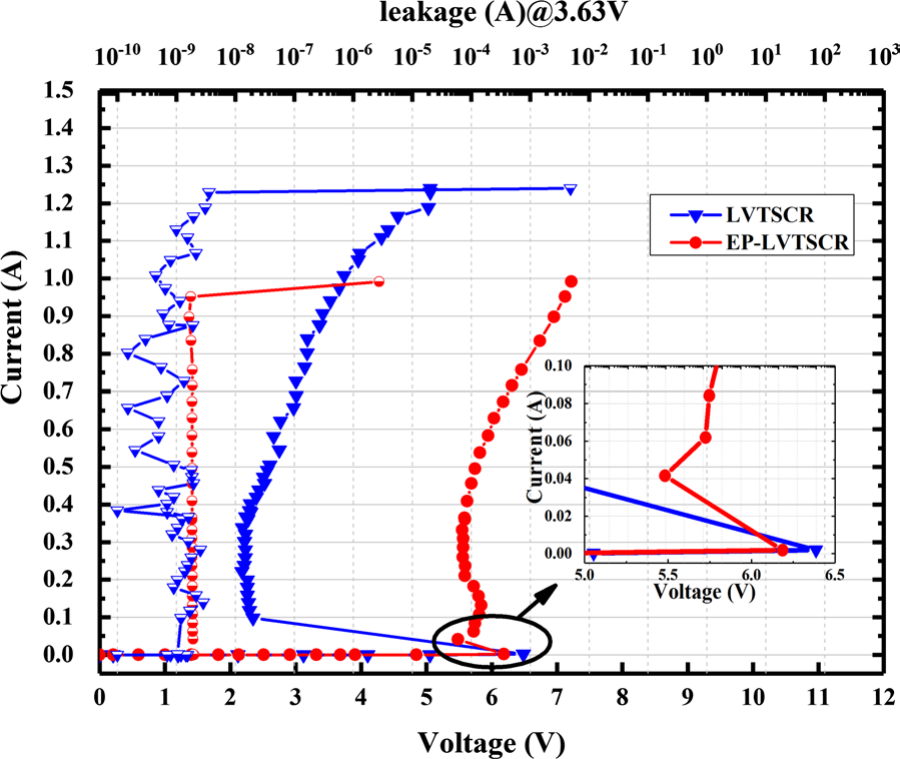
Measured TLP I–V and leakage currents of EP-LVTSCR and the conventional LVTSCR with same die area

The ESD characteristics of the conventional LVTSCR and EP-LVTSCR were measured using Hanwa TED-T5000 TLP tester with 10 ns rise time and 100 ns pulse width, and the leakage currents were measured under 3.63 V (1.1 * VDD) DC voltage bias after each TLP stress. Measured TLP I–V and leakage characteristics of the EP-LVTSCR and LVTSCR are shown in Fig. 5. Apparently, the EP-LVTSCR possesses a higher *V*_h_ of 5.49 V compared with the conventional LVTSCR of 2.18 V. Although the EP-LVTSCR has a significant improvement on the holding voltage, its *I*_t2_ just decreased by about 0.29 A, which benefits from the assistant of branch current path. Besides, EP-LVTSCR also performs a *V*_t1_ decreasing from 6.49 to 6.18 V. For 2.5 V or 3.3 V IO pin in 28-nm CMOS processes, the ESD design window ranged from 3.63 to 9.4 V with 10% safety margin consideration. Therefore, the proposed EP-LVTSCR can be used as a valid ESD protection solution for 2.5 V/3.3 V IO ports by overcoming the latch-up issue in a traditional LVTSCR-type structure.

**Fig. 6:**
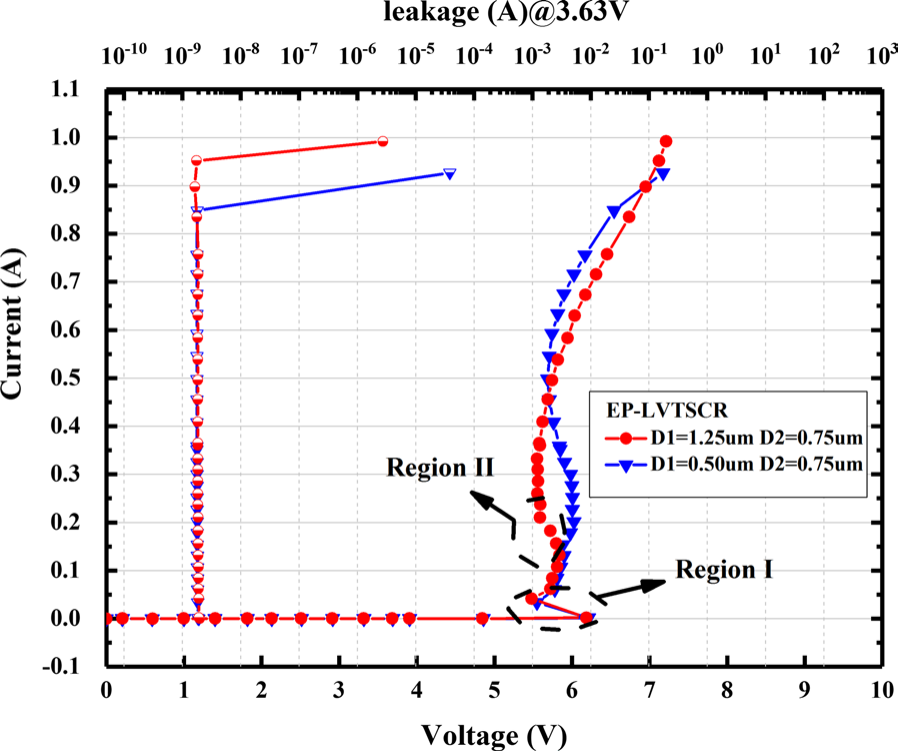
Measured TLP I–V and leakage currents of EP-LVTSCR with two different D1

In this article, the electrical characteristics of the proposed ESD protection were optimized by modifying the design variables D1 and D2. Figure 6 illustrates the TLP measurement results of EP-LVTSCRs with two different D1. It is noticed that the I–V curves of EP-LVTSCRs show two snapback regions due to the multi-triggering effects during turn-on of the devices. The first snapback region I suggests the conduction of the trigger path which is indicated in Fig. 2a, while the second snapback of region II is induced by the turn-on of the SCR path. When D1 decreases from 1.25 to 0.5 um, the *I*_t2_ decreases by about 0.1 A and the holding current in the second snapback is increased by about 0.17 A. This is because the well resistances *R*_NW2_ and *R*_PW2_ (Fig. 1) are decreased with the D1 diminution, hence requiring more current to trigger and sustain the conduction of SCR.

**Fig. 7:**
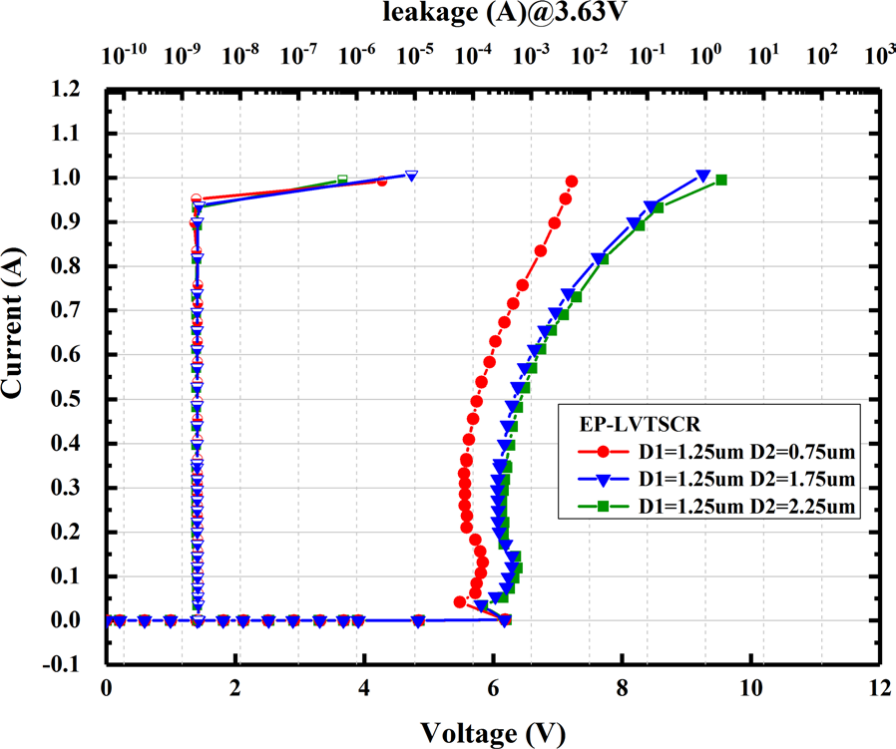
Measured TLP I–V and leakage currents of EP-LVTSCR with three different D2

Figure 7 shows the TLP I–V results of EP-LVTSCR with three different D2. When D2 increases from 0.75 to 2.25 µm, the ballast resistance of ND increased gradually, resulting in an increase in the resistance of the branch path, and further enlargement in *R*_on_, which can be observed by the slope variations of the IV characteristic curves in Fig. 7. As a result, the *V*_h_ elevates from 5.5 to 5.8 V with the D2 increase and without significant changes in I_t2_.

## Conclusions

An enhanced ESD device called the EP-LVTSCR has been designed and fabricated in a 28-nm CMOS technology. The mechanisms of the proposed devices were also demonstrated with TCAD simulations. Compared with the conventional LVTSCR, the proposed EP-LVTSCR possesses a lower trigger voltage of 6.2 V and a significantly higher holding voltage of 5.5 V due to its improved trigger mechanism and branch conduction effect. With such a higher increase in *V*_h_, the failure current of the EP-LVTSCR only decreased by about 20%. Besides, the proposed structure operates with a lower turn-on resistance as well as a reliable leakage current of about 2 nA at 3.63 V voltage, so it is highly applicable for protecting 2.5 V/3.3 V I/O pins. Furthermore, the EP-LVTSCRs are also expected to provide an ESD protection on 5 V power circuits with benefit from their adjustable holding voltage characteristics.

## Data Availability

All data generated or analyzed during this study are included in this published article.

## Abbreviations

ESD: Electrostatic discharge
SCR: Silicon-controlled rectifier
MLSCR: Modified lateral SCR
LVTSCR: Low-voltage-triggered SCR
CMOS: Complementary metal oxide semiconductor
IC: Integrated circuits
*V*_t1_: Trigger voltage
*V*_h_: Holding voltage
BJT: Bipolar junction transistors
*R*_on_: Turn-on resistance
*I*_t2_: Failure current
TLP: Transmission line pulse
TCAD: Technology computer-aided design
SAB: Silicide block
*V*_th_: Threshold voltage
DC: Direct current
